# Nutrition Outcomes and Interventions in Older People in Africa: A Systematic Umbrella and Scoping Review

**DOI:** 10.1093/nutrit/nuaf109

**Published:** 2025-07-04

**Authors:** Anthony Muchai Manyara, Tadios Manyanga, Shane Naidoo, Kate Mattick, Rudo Chingono, Grace M E Pearson, Opeyemi Babatunde, Niri Naidoo, Kate A Ward, Celia L Gregson

**Affiliations:** Global Health and Ageing Research Unit, Bristol Medical School, University of Bristol, Bristol BS10 5NB, United Kingdom; The Health Research Unit Zimbabwe, Biomedical Research and Training Institute, Harare, Zimbabwe; Health and Rehabilitation, University of Cape Town, Rondebosch, Cape Town, South Africa; The Health Research Unit Zimbabwe, Biomedical Research and Training Institute, Harare, Zimbabwe; Brighton and Sussex Medical School, Brighton BN1 9PX, United Kingdom; The Health Research Unit Zimbabwe, Biomedical Research and Training Institute, Harare, Zimbabwe; Global Health and Ageing Research Unit, Bristol Medical School, University of Bristol, Bristol BS10 5NB, United Kingdom; Older Persons Unit, Royal United Hospitals Bath NHS Foundation Trust, Bath BA1 3NG, United Kingdom; School of Medicine, Primary Care Centre Versus Arthritis, Keele University, Keele ST5 5BG, United Kingdom; Division of Physiotherapy, Department of Health and Rehabilitation Sciences, University of Cape Town, Cape Town, South Africa; MRC Unit The Gambia, London School of Hygiene and Tropical Medicine, Banjul, The Gambia; MRC Lifecourse Epidemiology Centre, Human Development and Health, University of Southampton, Southampton SO17 1BJ, United Kingdom; Global Health and Ageing Research Unit, Bristol Medical School, University of Bristol, Bristol BS10 5NB, United Kingdom; The Health Research Unit Zimbabwe, Biomedical Research and Training Institute, Harare, Zimbabwe

**Keywords:** nutrition, Africa, malnutrition, older people, healthy aging, obesity, vitamin D

## Abstract

Africa’s older population is rapidly increasing, necessitating the development of healthy aging interventions. Nutrition is a key component of healthy aging. Evidence synthesis on nutrition outcomes of older adults in Africa is emerging but a synthesis on interventions is lacking. The aim was to synthesize evidence from reviews on older people in Africa to determine the prevalence of nutrition outcomes and associated factors (phase 1) and implemented interventions (phase 2). Literature searches using Medline, EMBASE, Web of Science, African Index Medicus, and African Journals Online were conducted up to May 9, 2024. After screening, 25 reviews (for phase 1) and 22 articles (for phase 2) were selected for inclusion. Most reviews (n = 16; 64%) were systematic, with 8 having a meta-analysis, and published between 2020 and 2023 (n = 20; 80%). The pooled prevalence of malnutrition (being underweight) was 21% (evidence from 5 reviews), 26% for sarcopenia (1 review), 27% for obesity (3 reviews), 32% for constipation (1 review), 39% for food insecurity (2 reviews), 49% for dental caries (1 review), and 64% for vitamin D insufficiency and deficiency (2 reviews). The 22 articles on nutritional interventions represented only 6 countries, mostly South Africa (64%; 14/22), evaluated using randomized trials (n = 10; 45%) and educational interventions (n = 10; 45%). Reported interventions were not typically underpinned by supporting systematic reviews or a contextual evidence base, did not account for the minimally important clinical difference, lacked evidence of community engagement, and were not reported transparently. Nutritional research is needed on older adults outside of South Africa and beyond malnutrition. Future nutritional interventions (ideally, multicomponent) for older people in Africa should consider targeting the multiple nutritional and practical needs (eg, dietary counseling, supplementation) of older adults. Intervention development should be evidence-based, include engagement with older people, and follow complete and transparent reporting.

## INTRODUCTION

The number of older Africans (≥60 years) is projected to triple by 2050.^1^^,^^2^ This will result in increased multimorbidity and disability necessitating interventions^3^ that maximize healthy aging—that is, sustaining physical and mental capacities for people across the life course, to improve well-being in older age.^4^ Lifestyle factors (eg, physical activity, diet) are important determinants of morbidity, disability, healthy aging, and mortality. A recent scoping review has synthesized physical activity interventions among older people in Africa.^5^ Apart from physical activity, diet is an important determinant of aging.^6–8^ Healthy diets (eg, high in fiber and healthy oils and fats) are associated with longevity and better cardiometabolic, musculoskeletal, and cognitive health,^9^ while less-healthy diets (eg, high in sugar and salt, low in fiber) are important risk factors for noncommunicable diseases, including cancer and cardiovascular diseases.^10^ The nutritional needs of older people are different from those of the general population, and interventions must target a range of issues, including increasing overall food intake, protein intake, and micronutrient supplementation.^11^^,^^12^ Older adults’ opinions should inform such interventions about their nutritional needs,^13^ as well as local and contextual evidence to ensure cost-effectiveness.^14^

Understanding the nutritional needs of older African adults and interventions already implemented can inform future healthy aging interventions. However, to date, nutrition interventions in Africa have mainly focused on maternal and childhood nutrition. For example, a recent scoping review on barriers and facilitators to the implementation of nutrition interventions in Africa only included articles that focused on maternal and childhood nutritional interventions or general population nutritional interventions, with no consideration of nutrition interventions for vulnerable older adults.^15^ The skewed focus on maternal and childhood nutrition interventions was highlighted in an early narrative review published in 2001 on nutrition among older adults in Africa.^16^ More recently, emerging evidence has been published on the nutritional needs of older adults in Africa, including recent evidence syntheses on malnutrition,^17^^,^^18^ factors associated with nutrition,^19^ and food insecurity.^20^ These reviews have all called for urgent nutritional interventions for older people. With these emerging evidence syntheses, it is important and timely to review what has been reported to date; describe the prevalence of nutrition outcomes, dietary behavior and associated factors, and interventions already designed or evaluated; and identify gaps in the empirical research and inform future intervention development. Therefore, this article presents 2 interlinked evidence synthesis phases, both nutrition-specific (eg, supplementation) and nutrition-sensitive (eg, cash transfers), in older adults in Africa.

## METHODS

This review was preregistered in Open Science Framework,^21^ with the protocol published.^22^ It is reported according to Preferred Reporting Items for Systematic Reviews and Meta-Analyses for Scoping Reviews^23^ (see **File S1**). The review had 2 phases—phase 1: a review of reviews on nutrition in older people in Africa; and phase 2: a review of nutrition interventions in older people in Africa. We defined older adults as people aged 50 years and older. Studies were included if the mean age or 70% of the study population (signifying a sufficient majority) was 50 years or older. This age threshold reflects the lower life expectancy in Africa compared with high-income countries, where older adults are often defined using a higher age threshold.^24^

### Literature Search and Selection

For both review phases, 5 databases—Medline and EMBASE (via Ovid), Web of Science, African Index Medicus, and African Journals Online—were searched between November 28, 2023, and May 9, 2024. Searches were supplemented with Google Scholar searches for gray literature. Search strategies for both review phases were developed in consultation with an experienced systematic reviewer and librarian (see **File S2**). Search results from the Ovid and Web of Science databases were exported to EndNote for duplicate removal and then uploaded into Rayyan (Qatar Computing Research Institute, Doha, Qatar)^25^ for title, abstract, and full-text screening, which was conducted independently by 2 reviewers from a team of 4 (A.M.M., T.M., K.M., S.N.). Disagreements were resolved through group discussion. For efficiency, record screening in website-like databases, African Journals Online, African Index Medicus, and Google Scholar was conducted by the lead author (A.M.M.) using title and content text and, if needed, by accessing full texts.^26^ For phase 1, articles were included if they reported a review of any kind of synthesizing evidence on nutrition in older adults living in Africa. This included global reviews on older adults, with studies from Africa, as well as reviews on the general population in Africa that included studies and evidence on older adults. For phase 2, articles were included if they described any aspect of nutritional interventions (eg, type, design, implementation, evaluation) piloted or implemented among older adults in Africa (**Table 1**).

**Table 1.** PICOS Criteria for Inclusion and Exclusion of Studies

Parameter	Inclusion and exclusion criteria
Population	Older adults who are community-dwelling or residing in any long-term care facilities, such as nursing homes or retirement centers. Older adults are defined as those aged ≥50 years.^a^ Studies were included if the mean age or 70% of the study population was ≥50 years. The study had to be conducted, entirely or in part, in an African country.
Intervention	Any nutritional intervention or multicomponent interventions with a nutrition component. Studies were included if they described any of the findings relevant to the research questions, including intervention development process, piloting, feasibility testing, and evaluation of interventions.
Comparison	Any comparison, if available
Outcomes	Any outcome reported
Study design	Any study design Abstracts and theses were excluded

a An age of 50 years was used to define an older adult in Africa, given that life-expectancy is lower in the region compared with high-income countries, where an age of 60 or 65 years is traditionally used to define “older.”

### Data Extraction, Charting, and Synthesis

Data were extracted by 1 reviewer (A.M.M.), charted, and analyzed in Microsoft Excel (Microsoft Corporation, Redmond, WA, USA). Extracted data included characteristics of articles (eg, year of publication, country or region of focus, type of article or study design) and evidence that answered the research questions. Quantitative data were analyzed using either medians (and interquartile range) or counts and percentages, while qualitative data were analyzed using simple thematic analysis.^27^

#### Phase 1

Systematic reviews reported the prevalence of 7 nutrition and nutrition-related outcomes: malnutrition, obesity, food insecurity, vitamin D deficiency, sarcopenia, constipation, and dental caries. Malnutrition and obesity (including overweight and obese body mass index categories) reviews reported meta-analyses, and the meta-analyzed prevalences of each of these outcomes (ie, malnutrition and obesity) were separately combined as median and interquartile range. Reviews on vitamin D insufficiency (including deficiency), food insecurity, and dental caries did not report a meta-analysis; hence, the prevalences from individual relevant studies were combined as median (and interquartile range). One review each for sarcopenia and constipation had a meta-analysis, and the results were re-presented (with 95% CIs) without modification. The median (and interquartile range) number of studies included, countries represented, and total sample size of participants reported in meta-analyses and/or reviews were computed. Where only 1 review reported a nutrition outcome, the total number of studies included, countries represented, and the total sample size from individual studies were computed instead of medians. The medians (and interquartile range) were determined using R (version 4.3.1; R Foundation for Statistical Computing, Vienna, Austria).^28^ Factors associated with dietary behavior and nutrition outcomes were categorized into individual, social, physical, and macro-environmental factors based on an ecological framework by Story et al.^29^ To assess the quality of systematic reviews and meta-analyses, the following details were extracted: the proportion including 10 or fewer studies, searching for gray and unpublished literature, performing a quality assessment, using a reporting guideline, and reporting significant publication bias and heterogeneity.^30^ For reviews that reported outcomes of quality assessment, the median percentage (interquartile range) of studies reporting good or high quality, low bias, or a score equivalent to 80% of the overall quality score were computed.

#### Phase 2

The included studies were described using the following details: study design, country, participant sample size, sex and age, and intervention type (eg, educational, food aid). Data extracted on unique interventions (ie, considering that 1 intervention can result in multiple publications) included the disease or nutrition area targeted, intervention content and delivery, outcomes measured, what worked well (or did not), and the authors' conclusions. Intervention content included details on components (eg, supplement tablets, educational materials), while delivery included approach (eg, individual/group delivery), follow-up period, intensity (eg, daily intake, weekly intervention), setting (eg, community, health facilities), and personnel involved in the delivery. Outcomes used to evaluate nutrition interventions were extracted verbatim and categorized into biomarkers (eg, blood pressure), behavioral (eg, food intake), intermediate outcomes (eg, body mass index, malnutrition), and patient-important outcomes (eg, morbidity).^31^^,^^32^

Finally, the design, evaluation, and reporting quality of interventions was assessed by checking whether randomized controlled trials (RCTs) were supported by a cited systematic review,^33^ local evidence supporting the intervention was referenced, a theory was used for behavioral interventions, community or patient engagement was reported, minimal clinically important difference (smallest benefit of value to intervention users) was defined,^34^ and a relevant reporting guideline was used. Each was categorized as yes, no, or partial.

## RESULTS

**Figure 1.**
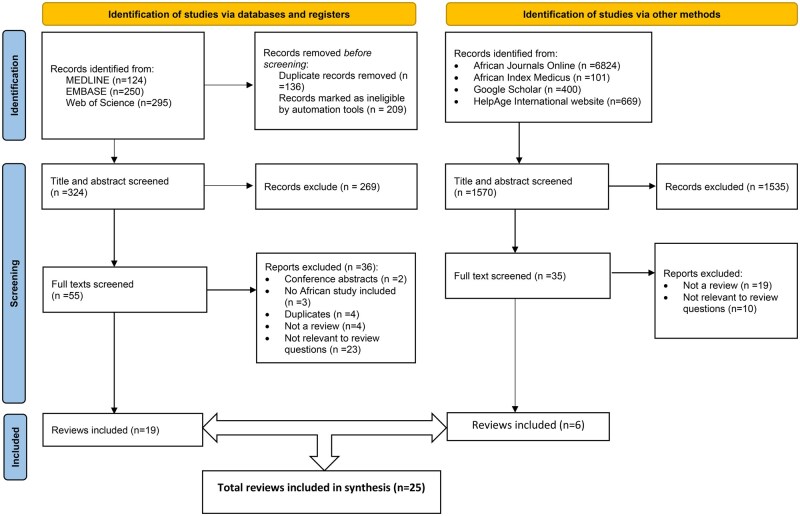
Literature Search and Inclusion Flow Diagram for Phase 1: Review of Reviews on Nutrition in Older People in Africa

**Figure 2.**
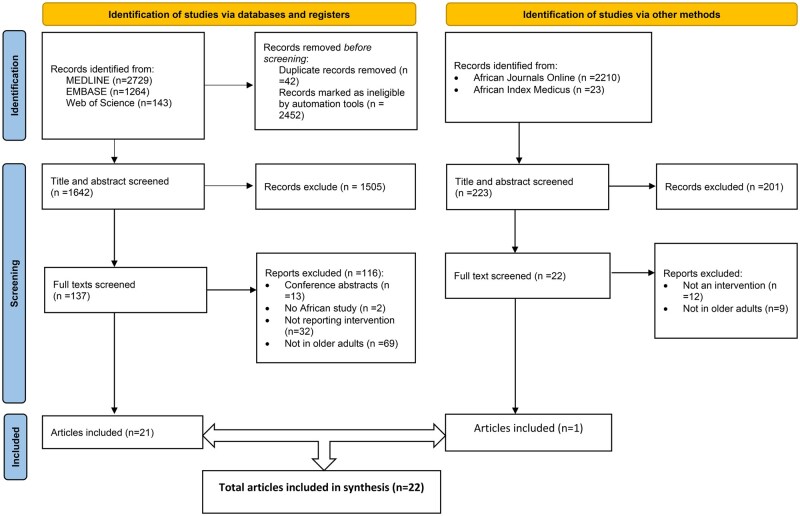
Literature Search and Inclusion Flow Diagram for Phase 2: Review of Nutrition Interventions in Older People in Africa

**Figures 1** and **2** show the identification, screening, and inclusion criteria of records for phases 1 and 2, respectively. After screening, 25 reviews on nutrition outcomes and 22 articles describing interventions were included.

### Phase 1: Review of Reviews

**Table 2** shows the characteristics of the 25 included reviews. Sixteen (64%) reviews were systematic, half of which (*n* = 8; 32%) included a meta-analysis. Twenty (80%) reviews were published between 2020 and 2023, 10 (40%) focused on Africa or sub-Saharan Africa (SSA), and 17 (68%) explicitly focused on older people. Five (of 16 systematic reviews; 31%) included 10 or fewer studies. Ten reviews (of 16; 63%) searched for gray or unpublished literature, while 75% (*n* = 12) performed a quality assessment. Of the 8 that reported on the outcome of the quality assessment, the median proportion (IQR) of included articles with high quality was 49% (43%–63%). Thirteen reviews (81%) used a reporting guideline, while 7 (44%) assessed publication bias. Of the 7, only 2 reviews (29%) found a significant publication bias. In the 7 meta-analyses that assessed heterogeneity, all found high levels of heterogeneity (>75%).

**Table 2.** Characteristics of the 25 Reviews Included in Phase 1: Review of Reviews on Nutrition in Older People in Africa

Characteristics	*n* (%)
Type of review	
Systematic with meta-analyses	8 (32%)
Systematic	8 (32%)
Scoping	3 (12%)
Other^a^	6 (24%)
Publication year	
2008–2019	5 (20%)
2020–2023	20 (80%)
Geographical focus of review	
Africa/SSA	10 (40%)
Region in Africa	3 (12%)
Specific countries in Africa	9 (36%)
Global review with African studies	3 (12%)
Representation of World Bank income category^35^	
Low	16 (64%)
Lower middle	18 (72%)
Upper middle	16 (64%)
Review explicitly on older people	
Yes	17 (68%)
No	8 (32%)
Topics covered^b^	
Malnutrition^c^	8 (32%)
Obesity	7 (28%)
Dietary behavior	4 (16%)
Food insecurity	2 (8%)
Factors associated with nutrition	10 (40%)
Micronutrient deficiency^d^	3 (12%)
Nutrition/aging policies	2 (8%)
Constipation	1 (4%)
Dental caries	1 (4%)
Sarcopenia	1 (4%)
Number of topics covered	
1	13 (52%)
≥2	12 (48%)

a Includes desk, rapid, and narrative reviews.

b Overlapping proportions.

c Malnutrition in reviews was defined using either the Mini Nutritional Assessment, body mass index, mid-upper arm circumference, or calf circumference.

d Vitamin D and zinc.

Abbreviation: SSA, sub-Saharan Africa.

**Table 3** shows the prevalence of nutrition outcomes and sources of evidence. The median prevalence of malnutrition, obesity, food insecurity, dental caries, and vitamin D insufficiency and deficiency was 21% (evidence from 5 reviews), 27% (3 reviews), 39% (2 reviews), 49% (1 review), and 64% (2 reviews), respectively. The pooled prevalence based on a single meta-analysis was 26% for sarcopenia^36^ and 32% for constipation^37^ (**Table 3**). The median number of studies included in the reviews ranged from 6 to 28, while the median number of countries represented ranged from 5 to 11 (**Table 3**).

**Table 3.** Prevalence of Nutrition Outcomes and Source of Evidence in Phase 1: Review of Reviews on Nutrition in Older People in Africa

	Median prevalence, % (IQR)	No. of reviews	Total studies included, median (IQR)	Total countries represented, median (IQR)	Total sample size for each review, median (IQR)
Malnutrition^a^	21% (18%-21%)	5	28 (14-32)	11 (1-13)	9611 (6573-12 804)
Obesity	27% (17%-33%)	3	13 (12-23)	8 (5-11)	375 (222-537)
Vitamin D insufficiency^b^	64% (30%-76%)	2	6 (4-8)	5 (3-6)	957 (615-1298)
Food insecurity^c^	39% (31%-62%)	2	19^d^	28^d^	502 (169-1200)
Dental caries	49% (46%-74%)	1	3^d^	3^d^	769^d^
Sarcopenia	26% (19%-33%)^e^	1	6^d^	5^d^	10 656^d^
Constipation	32% (22%-45%)^e^	1	2^d^	2^d^	514^d^

a Malnutrition in reviews was defined using either the Mini Nutritional Assessment, body mass index, mid-upper arm circumference, or calf circumference.

b Includes deficiency and insufficiency.

c One of reviews only had 1 relevant study so the number of studies and countries represented is a sum from the 2 reviews rather than median.

d Total number rather than median.

e Pooled prevalence (95% CIs) taken directly from reviews.

A systematic review in Nigeria, using a meta-regression epidemiologic model accounting for study sample size, study period, and age, estimated the prevalence of obesity to be 55% in people aged 50 years and older.^38^ A scoping review in Ghana reported the prevalence of underweight and overweight in people aged 50 years and older as 10% and 20%, respectively, with a high central obesity (67%) prevalence in those aged older than 60 years.^39^

#### Dietary Behavior

Four (of 25; 8%) reviews reported on dietary behaviors: 1 systematic review^40^ and 3 narrative reviews.^41–43^ One South African review reported that sugar intake was lower in those aged 65 years and older compared with younger age groups, although the authors noted that some older people may be consuming more than the recommended sugar intake.^41^ Two South African reviews found that adults aged 55 years and older were less likely to consume high-fat and street foods than those under 55 years.^40^^,^^41^ It was uncertain if the South African older adults met the recommended daily fat intake, with authors of 1 review arguing that, although it seemed the older people were within the recommended intake, the diets were generally low in oily fish, nuts, and vegetable oils.^41^ Another review noted that fat intake as a proportion of total calories was lowest in older Black people in South Africa.^42^ Fruit and vegetable intakes were reported to be low and below the recommended intake by 2 narrative reviews, based on evidence from SSA^42^ and from South Africa, Ghana, and Uganda.^43^ Salt intake was reported to be lower in older adults than in younger adults in South Africa,^40^^,^^41^ with 1 review reporting that 58% of those aged 65 years and older had actively lowered their salt intake.^41^ According to a narrative review on SSA, protein intake, particularly from dairy and animal sources, was reported to be low.^42^ The same review reported low micronutrient intake (vitamins, calcium, selenium, magnesium, copper, biotin) among Black South Africans.^42^

#### Factors Associated With Dietary Behaviors and Nutrition Outcomes

**Figure 3.**
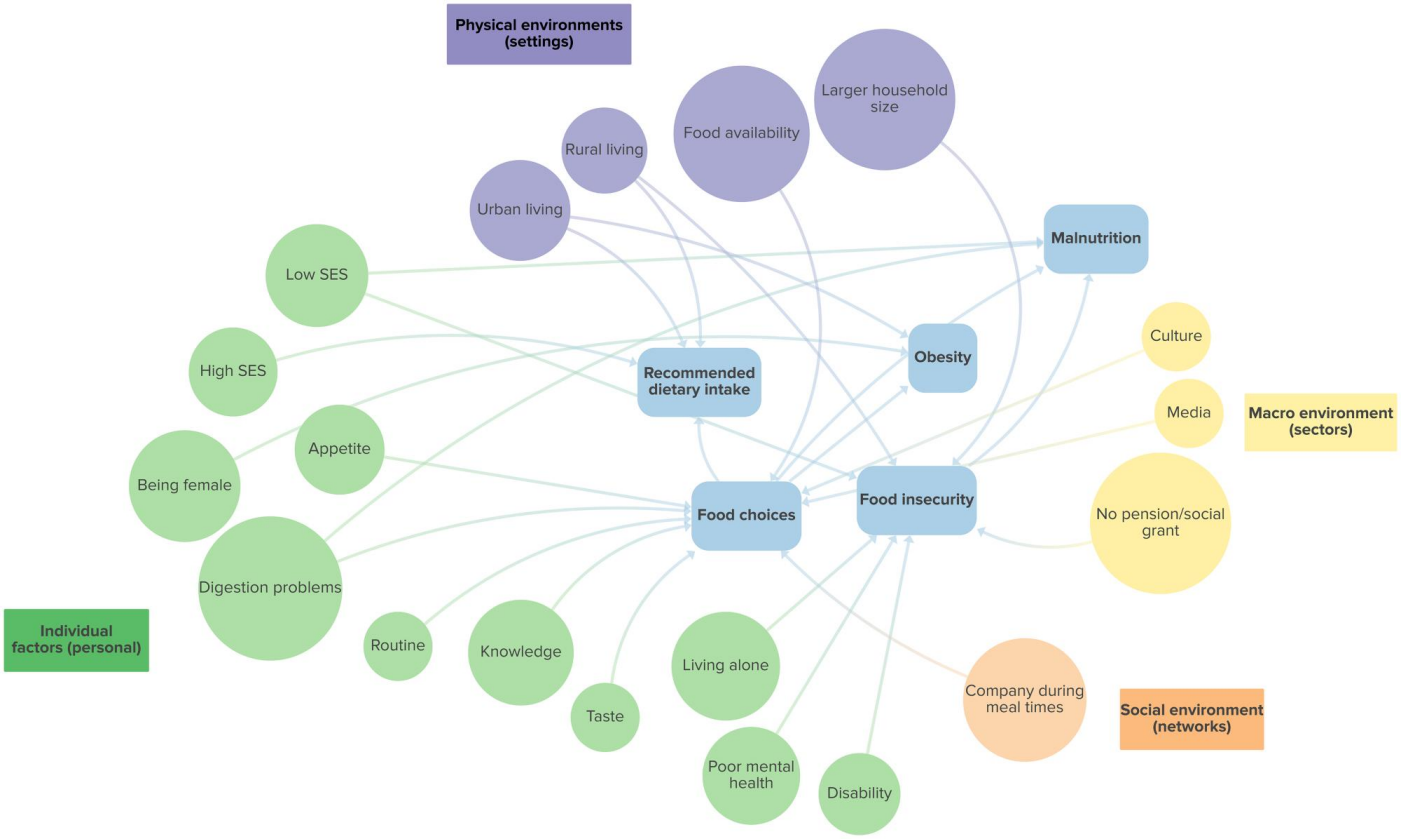
Factors Associated With Dietary Practices and Nutrition Outcomes Reported in 7 Reviews (Phase 1: Review of Reviews of Nutrition in Older Adults in Africa) Categorized Using an Ecological Framework by Story et al.^29^ Abbreviation: SES, socioeconomic status

Seven (of 25; 28%) reviews reported on factors associated with dietary behavior and nutrition outcomes: 3 systematic reviews,^20^^,^^44^^,^^45^ 2 scoping reviews,^19^^,^^46^ and 2 narrative reviews.^43^^,^^47^ The associated factors were categorized into individual, social, physical, and macro-environment factors. **Figure 3** summarizes these factors and their associations with recommended dietary intake, obesity, food insecurity, and malnutrition. **Table S1** shows the citations supporting the associations. Most of the factors (11/19, 58%) reported were at the individual level. Factors associated with more than 1 behavior or nutrition outcome were socioeconomic status, being female, having digestive problems, and rural/urban living (**Figure 3**).

#### Policies

Two reviews described policies related to older people in Ethiopia^48^ and nutrition and health in Ghana.^49^ Both reviews found that policy documents related to the nutrition of older people had not been written.

### Phase 2: Review of Interventions

**Table 4** shows the characteristics of the 22 included articles describing nutrition interventions. The studies reported were from only 6 countries, with the majority from South Africa (64%; 14/22). Ten (of 22; 45%) used an RCT study design, with sample sizes ranging from 48 to 1013 participants and a proportion of women of 50% or more. Most of the interventions described were educational (45%; 10/22) (see **Table 4**).

**Table 4.** Characteristics of 22 Included Articles in Phase 2: Review of Nutrition Interventions in Older People in Africa

Study, year of publication	Study country	Study design	Sample size, age, and study setting	Type of intervention
1. Cappuccio et al, 2006^50^	Ghana	RCT	1013 Participants (62% female) Mean age 55 y from rural and semi-urban settings	Educational
2. Charlton et al, 2008^51^	South Africa	RCT	92 Participants (84% female) Mean age 62 y, from an urban setting	Dietary modification and supplementation
3. Oldewage Theron and Kruger, 2009^52^	South Africa	Cohort intervention study	140 Older participants from an urban setting	Food aid
4. Bhurosy and Jeewon, 2013^53^	Mauritius	RCT	189 Participants (52% female) ≥82% Aged >50 y from urban settings	Educational
5. van Velden et al, 2015^54^	South Africa	RCT	100 Participants Mean age 65 y from an urban setting	Dietary supplementation
6. Muchiri et al, 2016^55^	South Africa	Qualitative process evaluation	41 Participants (88% female) Mean age 59 y from a rural setting	Educational
7. Muchiri et al, 2016^56^	South Africa	RCT	82 Participants (87% female) Mean age 59 y from a rural setting	Educational
8. Muchiri et al, 2016^57^	South Africa	RCT	82 Participants (87% female) Mean age 59 y from a rural setting	Educational
9. Shalaby et al, 2016^58^	Egypt	Quasi-experiment	115 Participants (53% female) Mean age 66 y from a rural setting	Educational
10. Lloyd-Sherlock et al, 2018^59^	South Africa	Pilot intervention and qualitative evaluation	20 Participants (% female not reported) 60+ y in rural settings	Dietary modification and educational
11. Napier et al, 2021^60^ 12. Napier et al, 2018^61^	South Africa	Qualitative case study	Phase 1: 86 Older adults (67% female) Mean age 67 y from rural and urban settings Phase 2: 123 Older adults (67% female) Mean age 69 y from rural and urban settings	Dietary guidelines
13. Charlton et al, 2021^62^	South Africa	Pre-post impact evaluation	1298 Participants (76% female) Mean age 54 y from rural and urban settings	Legislation
14. Muchiri et al, 2021^63^	South Africa	RCT	77 Participants (87% female) Mean age 57 y from an urban setting	Educational
15. Elsaid et al, 2021^64^	Egypt	RCT	80 Participants (50% female) 55+ y from an urban setting	Dietary supplementation
16. Kandhari et al, 2021^65^	Tanzania	RCT	48 Participants (81% female) Mean age 61 y from a rural setting	Dietary supplementation
17. Kuhn et al, 2022^66^	South Africa	RCT	57 (74% female) Mean age: 73 y in an urban setting	Dietary supplementation
18. Grobler et al, 2022^67^	South Africa	Quasi-experimental	104 Participants 60+ y from an urban setting	Dietary supplementation
19. Fouad et al, 2022^68^	Egypt	Quasi-experimental	60 Participants 60+ y from a rural setting	Educational
20. Muchiri et al, 2023^69^	South Africa	Qualitative process evaluation	50 participants (80% female) Mean age 57 y from an urban setting	Educational
21. Nkurunziza et al, 2023^70^	South Africa	Qualitative case study	10 Older adults (90% female) Mean age 77 y from an urban setting	Food aid
22. Seid and Babbel, 2023^71^	Ethiopia	Quasi-experiment	271 Participants (55% female) Mean age 73 y from an urban setting	Educational

Summary		
Country	Study design	Intervention types
South Africa (64%; 14/22) Egypt (14%; 3/22) Ethiopia (5%; 1/22) Ghana (5%; 1/22) Mauritius (5%; 1/22) Tanzania (5%; 1/22) World Bank income category^35^: Low-income (5%; 1/22) Lower-middle (23%; 5/22) Upper-middle (73%; 16/22) _	RCT (45%; 10/22) Qualitative studies (23%; 5/22) Quasi-experimental (18%; 4/22) Other evaluation studies (14%; 3/22)	Educational (45%; 10/22) Dietary modification/supplementation (27%; 6/22) Food aid (9%; 2/22) Dietary guidelines (9%; 2/22) Educational and dietary modification (9%; 2/22) Legislation (5%; 1/22)

Abbreviation: RCT, randomized controlled trial.

The 22 articles described 15 unique interventions; **Table 5** shows the characteristics of these interventions. Four (of 15; 27%) intervened on hypertension and 4 on nutrition status (eg, malnutrition), 2 (13%) intervened on osteoporosis/arthritis, and 2 on diabetes; and 1 study addressed each of (7%) cardiovascular disease, influenza, and cognition. In the 14 interventions reporting delivery approach and setting, most were delivered at an individual (57%; 8/14) rather than a group level (29%; 4/14). Most were delivered in the community (57%; 8/14) rather than in facilities such as health or care centers (43%; 6/14).

**Table 5.** Description of the 15 Unique Tested Interventions in Phase 2: Review of Nutrition Interventions in Older People in Africa

Study, year of publication, country, study design	Type of intervention	Disease/nutrition area targeted	Intervention description	Intervention delivery approach, intensity, duration, and follow-up period	Setting delivered and personnel delivering
Cappuccio et al, 2006, Ghana, RCT^50^	Educational	Hypertension	1-h education session using flip charts	Group sessions Daily first week of the study and once weekly thereafter	Community Community health workers
Charlton et al, 2008, South Africa, RCT^51^	Dietary modification/supplementation	Hypertension	Brown bread, margarine, stock cubes, soup mixes and aromatic flavor enhancer with modified sodium, potassium, magnesium, and calcium content A salt replacement (Solo, Low Sodium Sea Salt Company Limited, Kent, UK) and 500 mL of maas (fermented milk commonly eaten)	Individual intervention daily 8-wk follow-up	Community Field workers
Oldewage Theron and Kruger, 2009, South Africa, cohort intervention study^52^	Food aid	Nutrition status	Provision of breakfast and lunch meals based on a culturally acceptable meal plan drawn up by the research team	Group provision of food 5 days weekly 2-y follow-up	Care center 12 Volunteers
Bhurosy and Jeewon, 2013, Mauritius, RCT^53^	Educational	Osteoporosis	2-h session consisting of: Presentations and discussions Participants experience sharing Use of posters/pamphlets Writing down calcium sources and action points Demonstration of simple exercises	Group sessions Six sessions 2-mo follow-up	Community Personnel delivering not reported
van Velden et al, 2015, South Africa, RCT^54^	Dietary supplementation	Osteoarthritis	7.5-g Supplement containing magnesium hydrogen phosphate 244 mg, calcium citrate 145 mg, potassium bicarbonate 783 mg, magnesium citrate 315 mg, potassium citrate 870 mg, di-calcium-phosphate 2-hydrate 973 mg, organic plant calcium, acerola extract and mannitol, and delivers ∼250 mg of elemental calcium	Individual Twice-daily intake 56-d follow-up with crossover on day 28	Community Physicians
Muchiri et al, 2016^56^ Muchiri et al, 2016^57^ South Africa, RCT	Educational	Diabetes	Educational materials (pamphlet and wall/fridge poster) Educational sessions Discussions and reflections Food displays and meal tasting Meal planning and goal setting Costing meals Portion size demonstration and practice Cooking demonstration and group cooking Vegetable gardening demonstration	Group sessions: 8 weekly 2-2.5-h sessions 4 monthly 1.5-h follow-up sessions 2 bimonthly 1.5-h follow-up sessions Total 26.5 h contact time per group 12-mo follow-up	Health facility Team of 3 dietitians and a horticulture officer
Shalaby et al, 2016, Egypt, quasi-experimental^58^	Educational	Nutrition status	Counseling sessions	Details not reported	Not reported
Muchiri et al, 2021, South Africa, RCT^63^	Educational	Diabetes	Educational materials (pamphlet and wall/fridge poster) and workbook Self-evaluation of laboratory results Educational sessions Discussions and reflections Food displays and meal tasting Group discussions Costing meals Portion-size demonstration and practice Individual counselling and goal setting	Group and individual sessions: 7-monthly 2-2.5-h group training sessions 1 Individual 15-30 min counseling and goal-setting session Bimonthly group follow-up sessions 12-mo follow-up	Health facility 2 Dietitians
Kandhari et al, 2021, Tanzania, RCT^65^	Dietary supplementation	Hypertension	Nitrate-rich beetroot juice and/or folate capsule	Individual Daily intake 60-d follow-up	Community Research team
Elsaid et al, 2021, Egypt, RCT^64^	Dietary supplementation	Influenza	Natural arabinoxylan rice bran product called Biobran/MGN-3, which is an immunomodulator	Individual Biobran/MGN-3 (supplement) 500 mg/d 3-mo follow-up	Health facility Health workers
Charlton et al, 2021, South Africa, pre-post impact evaluation^62^	Legislation	Hypertension	Legislation for mandatory maximum sodium levels in processed-food categories (eg, bread, cereal, salty snacks, processed meat)	Group (population) level 3-y follow-up	Community Government and industry
Fouad et al, 2022, Egypt, quasi-experimental^68^	Educational	Nutrition status	Nutrition booklet	Individual Duration and intensity not reported	Community Personnel not reported
Grobler et al, 2022, South Africa, quasi-experimental^67^	Dietary supplementation	Cardiovascular disease	Supplement containing >125% RDA of vitamin B_12_ (25 μg), vitamin B_6_ (50 mg), and folate (400 μg)	Individual Daily intake 6-mo follow-up	Day care center Fieldworkers
Kuhn et al, 2022, South Africa, RCT^66^	Dietary supplementation	Cognition	Two 410-g cans of pilchards and 75 g of fish paste	Individual Weekly intake 12-wk follow-up	Retirement center Research team
Seid and Babbel, 2023, Ethiopia, quasi-experimental^71^	Educational	Nutrition status	Flyers as a reminder to lead healthy lifestyle Nutrition counseling	Individual counseling 45-60-min sessions once per week 2-mo follow-up	Community 8 Trained nurses

Totals		
Intervention target	Delivery approach	Setting delivered
Hypertension (27%; 4/15) Nutrition status (27%; 4/15) Osteoporosis/arthritis (13%; 2/15) Diabetes (13%; 2/15) Cardiovascular disease (7%; 1/15) Influenza (7%; 1/15) Cognition (7%; 1/15)	Individual (57%; 8/14) Group sessions (29%; 4/14) Population level (7%; 1/14) Individual and group (7%; 1/14)	Community (57%; 8/14) Health facility (21%; 3/14) Care/retirement centers (21%; 3/14)

Abbreviations: RCT, randomized controlled trial; RDA, Recommended Dietary Allowance.

**Table 6** shows the intervention aims, outcome measures, and authors’ conclusions. Nine of 15 (60%) aimed to prevent disease; only 13% (*n* = 2) evaluated outcomes identified as important to patients; and most (80%, *n* = 12) found the tested intervention to be efficacious, as shown in **Table 6**.

**Table 6.** Description of Intervention Types, Outcomes Measured, and Conclusions From the 15 Tested Interventions in Phase 2: Review of Nutrition Interventions in Older People in Africa

Study, year of publication, country, study design	Type of intervention and target	Intervention aim	Type of outcome measured	Outcomes measured	Authors’ conclusions
Cappuccio et al, 2006, Ghana, RCT^50^	Educational for hypertension	Prevention	Biomarkers	Urinary sodium excretion and blood pressure levels	Intervention resulted in small reduction in both systolic and diastolic blood pressure but no effect on urinary sodium excretion
Charlton et al, 2008, South Africa, RCT^51^	Dietary modification/supplementation for hypertension	Treatment	Biomarkers	Resting office blood pressure level (primary) Average 24-h ambulatory systolic and diastolic blood pressure and awake and asleep blood pressure (secondary)	Intervention lowered blood pressure by a clinically significant magnitude
Oldewage Theron and Kruger, 2009, South Africa, Cohort intervention study^52^	Food aid for nutrition status	Prevention	Behavioral	Dietary diversity using dietary diversity scoring, food variety scoring and nutrient adequacy ratios	Intervention effective to improve short-term nutrition through improved the level of dietary diversity
Bhurosy and Jeewon, 2013, Mauritius, RCT^53^	Educational for osteoporosis	Prevention	Biomarkers and behavioral	Calcium intake, Health Belief scale scores, knowledge scores, and physical activity level (PAL)	Intervention effective in improving the dietary calcium intake, knowledge, self-efficacy, and PAL
van Velden et al, 2015, South Africa, RCT^54^	Dietary supplementation for osteoarthritis	Treatment	Patient-important outcome	Clinical signs and symptoms of osteoarthritis, ie, pain, tenderness, and stiffness of interphalangeal and metacarpophalangeal joints of the hand	Intervention efficacious and safe as sole therapeutic intervention, significantly reducing signs and symptoms of the hands
Shalaby et al, 2016, Egypt, quasi-experimental^58^	Educational for nutrition status	Prevention	Behavioral	Knowledge, attitudes, and dietary practices	Intervention effective in improving knowledge and changing attitudes to leading to improvements in dietary practices
Muchiri et al 2016^56^ Muchiri et al 2016^57^ South Africa, RCT	Educational for diabetes	Treatment	Biomarkers, intermediate, and behavioral	HbA1c (primary outcomes) BMI, blood pressure, blood lipids, changes in diabetes knowledge and the attitudes towards diabetes and its treatment (secondary outcomes)	Intervention not efficacious on HbA1c but improved specific dietary behaviors
Muchiri et al, 2021, South Africa, RCT^63^	Educational for diabetes	Treatment	Biomarkers, intermediate, and behavioral	HbA1c (primary outcomes) BMI, blood pressure, blood lipids, changes in dietary behaviors, knowledge, and diabetes management self-efficacy (secondary outcomes)	Intervention had limited effects on HbA1c, targeted dietary behaviors and behavior mediators but had positive effects on blood pressure
Kandhari et al, 2021, Tanzania, RCT^65^	Dietary supplementation for hypertension	Treatment	Biomarkers	Plasma nitrate and folate concentrations (primary outcomes) Changes in 24-h ambulatory blood pressure, whole-body nitric oxide production, serum homocysteine, C-reactive protein, nitro-tyrosine and salivary nitrite (secondary outcomes)	Acceptability of the interventions was high; self-reported compliance to the interventions was >90%
Charlton et al, 2021, South Africa, pre-post impact evaluation^62^	Legislation for hypertension	Prevention	Biomarkers and behavioral	Urinary sodium excretion and blood pressure Salt intake	Intervention significantly reduced overall salt intake by 1.15 g/d
Elsaid et al, 2021, Egypt, RCT^64^	Dietary supplementation for influenza	Prevention	Patient-important outcome	Incidence of infection (primary) Incidence of any clinically reported or laboratory-observed adverse effect (secondary)	Intervention enhanced the innate immune response and reduced influenza incidence
Fouad et al, 2022, Egypt, quasi-experimental^68^	Educational for nutrition status	Prevention	Intermediate and behavioral	Nutritional status was measured by the Mini-Nutritional Assessment (MNA) and dietary practices	Intervention effective in improving dietary habits and nutritional status
Grobler et al, 2022, South Africa, quasi-experimental^67^	Dietary supplementation for CVD	Treatment	Biomarkers and behavioral	Serum homocysteine, vitamins B_6_ and B_12_, folate levels, red blood cell count, mean cell volume, hemoglobin, hematocrit, and the nutritional intake of vitamin B_6_ and B_12_ and folate	Intervention reduced hyper-homocysteinemia translating to reduction in CVD risk
Kuhn et al, 2022, South Africa, RCT^66^	Dietary supplementation for cognition	Prevention	Intermediate	Cognition using the Cognitive Abilities Screening Instrument (CASI)	Intervention may improve the cognition of cognitively intact, resource-limited older people.
Seid and Babbel, 2023, Ethiopia, quasi-experimental^71^	Educational for nutrition status	Prevention	Intermediate and behavioral	Nutritional status was measured using a validated Mini Nutritional Assessment tool and dietary practices using the 24-h dietary recall, meal frequency, and dietary diversity score	Intervention could promote perception, diversify dietary consumption, and reduce the risk of undernutrition

Summary		
Intervention aim	Outcomes measured	Summary of results
Prevention (60%; 9/15) Treatment (40%; 6/15)	Biomarkers (20%; 3/15) Behavioral (13%; 2/15) Patient-important outcomes (13%; 2/15) Intermediate outcomes (7%; 1/15) Combination of biomarkers/intermediate outcomes and behavioral (46%; 7/15)	Positive intervention effects (80%; 12/15) Null intervention effects on some outcomes (20%; 3/15)

Abbreviations: BMI, body mass index; CVD, cardiovascular disease; HbA1c, glycated hemoglobin; RCT, randomized controlled trial.

**Table 7** shows the design, evaluation, and reporting characteristics of the 15 unique interventions. Most interventions (80%; 12/15) did not cite local supporting evidence, while the majority of RCTs (56%; 5/9) did not cite a systematic review to justify the development of their intervention. Five of the 9 (56%) behavioral interventions used a theory for intervention development. Few studies (27%; 4/15) reported on community or patient engagement. Most did not use a minimally clinically important difference (80%; *n* = 12) or a reporting guideline (87%; *n* = 13) (see **Table 7**).

**Table 7.** Design and Reporting of the 15 Interventions in Phase 2: Review of Nutrition Interventions in Older People in Africa

Study, year of publication, country, study design	Systematic review cited for RCTs	Local evidence cited	Behavioral theory used	Community/patient engagement reported	MCID used or reported	Reporting guideline used
Cappuccio et al, 2006, Ghana, RCT^50^	No	Yes	No	Yes	No	No
Charlton et al 2008, South Africa, RCT^51^	No	No	NA	No	No	No
Oldewage Theron and Kruger, 2009, South Africa, cohort intervention study^52^	NA	Yes	Yes	Yes	Partially	No
Bhurosy and Jeewon, 2013, Mauritius, RCT^53^	Yes	No	Yes	No	No	No
van Velden et al, 2015, South Africa, RCT^54^	No	No	No	No	No	No
Shalaby et al, 2016, Egypt, quasi-experimental^58^	NA	No	Yes	No	No	Yes
Muchiri et al 2016^56^ Muchiri et al 2016^57^ South Africa, RCT	Partially	No	Yes	Yes	No	No
Muchiri et al, 2021, South Africa, RCT^63^	Yes	No	Yes	Yes	No	No
Charlton et al, 2021, South Africa, pre-post impact evaluation^62^	NA	No	NA	No	No	No
Elsaid et al, 2021, Egypt, RCT^64^	No	Yes	NA	No	Partially	No
Kandhari et al, 2021, Tanzania, RCT^65^	Yes	No	NA	No	No	Partially
Fouad et al, 2022, Egypt, Quasi-experimental^68^	NA	No	No	No	No	No
Grobler et al, 2022, South Africa, Quasi-experimental^67^	NA	No	NA	No	No	No
Kuhn et al, 2022, South Africa, RCT^66^	No	No	NA	No	Yes	No
Seid and Babbel, 2023, Ethiopia, Quasi-experiment^71^	NA	No	No	No	No	NA
Total	No (56%; 5/9) Partially (11%; 1/9) Yes (33%; 3/9)	No (80%; 12/15) Yes (20%; 3/15)	No (44%; 4/9) Yes (56%; 5/9)	No (73%; 11/15) Yes (27%; 4/15)	No (80%; 12/15) Partially (13%; 2/15) Yes (7%; 1/15)	No (87%; 13/15) Partially (7%; 1/15) Yes (7%; 1/15)

Abbreviations: MCID, minimal clinically important difference; NA, not applicable; RCT, randomized controlled trial.

**Table 8** presents process evaluation findings from 5 interventions. All were from South Africa and used focus group discussions, with 3 supplemented by literature reviews, stakeholder discussions, questionnaires, and/or in-depth interviews. What worked well included fast delivery (ie, no need to wait in line to receive low-sodium salt and health education on hypertension at pension pay points, compared with waiting in line for services in health facilities)^59^; easy-to-follow education materials, group discussions, appropriate number and duration of education sessions, and reimbursement to attend sessions for an educational intervention^55^; and provision of inexpensive and tasty meals for a food aid intervention.^70^ Recommendations included the translation of educational materials into local languages,^55^^,^^60^^,^^61^ use of peer trainers,^69^ and family members of patients joining educational sessions.^69^

**Table 8.** Process Evaluation Themes of 5 Interventions in Phase 2: Review of Nutrition Interventions in Older People in Africa

Study, publication year, country	Intervention description and data-collection methods	What worked well?	What did not work well?	Recommendations
Muchiri et al, 2016,^55^ South Africa	Nutrition education for diabetes patients including the provision of education materials, demonstrations, and education sessions Self-administered, open-ended questionnaire and 5 focus-group discussions	Participants enjoyed perceived benefits, and satisfied with its content and delivery Education materials (wall or fridge poster, pamphlet) useful reminder for positive behavior for whole family Facilitators respectful approach Reimbursement of transport cost for meetings	—	Education materials should both be available in both English and the local language Open education sessions to family members Repeat all or some topics that were taught
Lloyd-Sherlock et al, 2018,^59^ South Africa	Provision of monthly low-sodium salt at pension delivery points and education about hypertension, blood pressure measurement, referral to primary care services 2 Focus-group discussions	High retention and continued engagement with intervention Fast delivery: intervention delivered at pension points meant no need to queue for long hours like in health center Stakeholders in support of intervention and open to working together	Participants could not find or afford fresh fruits and vegetables despite knowledge on their health benefits Ethical concerns on sustainability of the intervention leading to withdrawal of provision of free low-sodium salt	Work with local businesses to supply affordable low-sodium salt
Napier et al, 2018^61^ Napier et al, 2021^60^ South Africa	Food-based dietary guidelines for the elderly in South Africa Literature review, stakeholder discussions, expert panel input, and focus-group discussions	Translated guidelines were understood by the majority	English guidelines were only understood by English-speaking participants	Guidelines should be available in all South African official languages Strategy for implementation and evaluation of guidelines should be developed Guidelines should be used in the Integrated Nutrition Programme and form basis for nutrition education for elderly in South Africa
Muchiri et al, 2023,^69^ South Africa	Adapted nutrition for diabetes patients including the provision of education materials, demonstrations, and education sessions 5 Focus-group discussions	Participants enjoyed the intervention, gained knowledge and skills, and perceived benefits Participants satisfied with education materials, meeting and education session frequency and length	Diabetes medication not addressed in education sessions	Peer trainers for future interventions Reinforce information through revised content Family members to join some sessions Incorporate more medical content in sessions
Nkurunziza et al, 2023,^70^ South Africa	Meals on Wheels Community Service (MOWCS) provides readymade home meal deliveries for older people 10 Semi-structured interviews and 1 focus-group discussion	Access to inexpensive, healthy, and balanced meals that were well prepared and tasteful Good service from trained chefs and cooks	Taste was bland for some participants Financial and operation challenges, eg, short staffing and lack of kitchen and service delivery equipment Only social service operating in a vast area Relies on volunteers whose service can be unreliable Inability to expand intervention to wider community as it served mainly White women	Extending the intervention to all South African demographics to alleviate food insecurity in older adults

## DISCUSSION

This article presents a comprehensive synthesis of nutritional evidence on older adults in Africa, including the nutritional interventions that have been evaluated. Evidence synthesis of nutrition in older adults in Africa has increased in the last 4 years. The pooled prevalence of malnutrition in older adults was 21% based on 5 reviews, 26% for sarcopenia (1 review), 27% for obesity (3 reviews), 32% for constipation (1 review), 39% for food insecurity (2 reviews), 49% for dental caries (1 review), and 64% for vitamin D insufficiency (2 reviews). Dietary behavior and nutritional outcomes (malnutrition and obesity) were associated with individual factors (eg, gender, socioeconomic status), the physical environment (eg, rural/urban living), social environment (eg, company during meals), and macro-environment (eg, culture, media, pension grants). Most nutritional interventions have been educational, implemented in South Africa, evaluated using RCTs, targeted towards specific diseases (eg, hypertension, diabetes, arthritis), and delivered using an individualized approach in community settings. Only a few interventions have been evaluated using patient-centered outcomes, supported by systematic reviews or local-level evidence, and involved patient and community engagement in their design and/or conduct. Furthermore, none were reported using a suitable reporting guideline.

Compared with global meta-analyses that have included older adults, the pooled prevalences reported in this review are as follows: higher for malnutrition (21% vs 17%)^72^ and constipation (32% vs 19%),^37^ similar for sarcopenia (26% vs 10%–27%)^73^^,^^74^ and dental caries (49% vs 45%–49%),^75^^,^^76^ and lower for vitamin insufficiency (64% vs 74%)^77^ and obesity (27% vs 40%).^73^ Furthermore, the pooled prevalence of food insecurity identified in our review is higher than the pooled analysis of World Health Organization (WHO) Study on global AGEing and adult health (SAGE) data (from China, Ghana, India, Mexico, Russia, and South Africa)—39% vs 12%.^78^ The prevalence comparisons must be interpreted with caution given that data for vitamin D insufficiency, sarcopenia, dental caries, and constipation are each based on fewer than 10 studies. Furthermore, only 49% of included articles were rated to be of high quality and with low risk of bias, only 44% of the systematic reviews assessed publication bias, and all of the meta-analyses found high levels of heterogeneity. Notwithstanding, these findings imply the need for more research and evidence synthesis on other nutrition aspects beyond malnutrition, the latter having been the subject of 5 reviews published between 2022 and 2023. Furthermore, these findings suggest that nutritional interventions for older adults in Africa should be multicomponent and aimed at the following: promoting food security, healthy diets, healthy body weight, dietary supplementation, micronutrient sufficiency, and oral health. However, findings from synthesis of implemented interventions (phase 2 of this review) showed that most interventions were only educational and targeted specific diseases (eg, hypertension, diabetes, arthritis), showing a mismatch between local and current evidence on older adults’ nutritional needs and intervention development. Furthermore, there are no policy documents on nutrition in older adults in Africa to guide practice.

Intervening through education alone ignores the wider social and environmental determinants of health outcomes and behavior,^8^^,^^79^ such as the high prevalence of food insecurity observed in the current review. Furthermore, there is conflicting evidence on the effectiveness of nutrition educational interventions in older adults. A meta-analysis on this topic found that supplementation, environmental, and organizational interventions (to provide and improve food intake) rather than educational interventions were associated with improved nutritional and functional outcomes and prevention of fractures and falls,^80^ while another review found that supplementing nutrition education with other interventions, such as exercise and/or social skills, was associated with improved functional outcomes in older adults.^81^ Moreover, a pooled analysis of data in 9 RCTs found that dietary counseling combined with oral nutrition supplements was the most effective intervention for older adults at risk of malnutrition.^82^ Taken together, these findings imply the need for multicomponent interventions that combine various nutritional components (eg, dietary counseling, supplementation) and non-nutritional components, such as exercise. Such interventions should target multiple nutritional needs of older adults rather than specific health conditions, providing a more comprehensive and holistic intervention for older adults who seldom have just 1 isolated health condition.

With regard to intervention delivery, this review found that most interventions were delivered using an individual approach. While individual delivery approaches are justified for some interventions, such as food aid or supplementation, incorporation of group delivery may be ideal for educational interventions, or for changing cooking habits for a household. In the current review, half of the educational interventions were delivered through group sessions, with one using both group and individual approaches. Such a combined delivery allows for multicomponent interventions and leverages the benefits of an individual approach, such as personal goal setting,^83^^,^^84^ while group sessions allow for older people to learn from and support each other and form social bonds, among other benefits.^8^^,^^85^ Delivery of multicomponent interventions with group and individual sessions may be costly, financially and in terms of human resources, and could be made more efficient through using peers, rather than health workers, as stated in one of the recommendations arising from a process evaluation.^69^

Finally, and unfortunately, only a few interventions included in this review were evaluated using patient-centered outcomes, supported by relevant systematic review or local evidence, including patient and community engagement, and used a relevant reporting structure, while almost half of behavioral interventions used a relevant theory. These findings are consistent with those of a recent global review on group-based nutritional interventions in older adults, which found that most reported interventions had a high or unclear risk of bias and were not informed by a behavioral theory.^85^ Reference to systematic review evidence by RCTs provides a scientific and ethical justification for the intervention,^33^ while citation of relevant local evidence ensures contextual relevance, maximizing potential future scalability^14^—with both reducing research waste. Use of patient-centered outcomes^31^ and minimal clinically important differences^34^ ensure that interventions are beneficial to users, while utilizing behavioral theories and standardized reporting guidelines, such as the Template for Intervention Description and Replication (TIDieR)^86^ and CONSORT (Consolidated Standards of Reporting Trials),^87^ enable study replication and standardization.^84^^,^^85^^,^  ^88^ Participant and community engagement and involvement in research have multiple benefits, including improving the contextual relevance of research questions and study design, increasing participant recruitment and research impact.^13^^,^  ^89–91^ Therefore, it is important that all stakeholders, including researchers, funders, journal editors, and peer reviewers, pay attention to intervention outcomes, design, development process, and reporting to ensure effectiveness, uptake, replicability, and reduced research waste.

The strength of this review lies in its comprehensive synthesis of all available evidence on nutrition (and related concepts, such as oral health and sarcopenia) in older adults in Africa, including evaluated interventions. However, the diversity in the African continent and the low numbers of studies included in some reviews (phase 1) and only 6 countries represented in the evaluated interventions (phase 2) limit the generalizability of findings to the African region. In particular, for phase 2, there was low representation from low-income and lower-middle-income countries based on the World Bank income categories.^35^ Further, some prevalences, such as sarcopenia, may have been underestimated, given that there is no validated African definition.

Recommendations

- Nutritional intervention development informed by local and current evidence on older adults’ nutritional needs
- Develop multicomponent interventions that combine various nutritional components (eg, dietary counseling, supplementation) and non-nutritional components such as exercise
- Interventions evaluated using patient-centered outcomes
- Consider delivery of intervention through using peers rather than health workers, where appropriate
- Pay attention to intervention design, development process, and reporting to ensure effectiveness, uptake, replicability, and reduced research waste.
- Development of policy documents for nutrition in older adults
- More nutrition research on older adults outside of South Africa and beyond malnutrition

## CONCLUSION

There is a high prevalence of malnutrition, sarcopenia, obesity, constipation, food insecurity, dental caries, and vitamin D insufficiency among older adults in Africa. Of the few nutritional interventions researched in this population, most have been educational, from South Africa, evaluated using RCTs, targeted individual diseases (eg, hypertension, diabetes, arthritis), and delivered using an individual approach in community settings. Few intervention studies have been optimally developed and reported. Future nutritional interventions in older people in Africa should be multicomponent, combining various nutritional components (eg, dietary counseling, supplementation) and non-nutritional components such as exercise to target the multiple nutritional and functional needs of older adults and ideally delivered using individual- and group-based approaches. The development of these interventions should be evidence-based and include older people and communities in the design and conduct of the research, with results reported following standardized reporting guidelines. Finally, there is a need for nutrition research on older adults beyond South Africa and beyond addressing malnutrition.

## Supplementary Material

nuaf109_Supplementary_Data

## Data Availability

All data is provided in the results or supplementary material.
