# Supplementary material for: Nutrition Outcomes and Interventions in Older People in Africa: A Systematic Umbrella and Scoping Review
Source: Nutr Rev. 2025 Jul 4;84(5):1051–69. doi: 10.1093/nutrit/nuaf109 (PMC13075486; doi:10.1093/nutrit/nuaf109)
Supplement: nuaf109_Supplementary_Data [file nuaf109_supplementary_data.zip › Supplementary File 2 2024.11.06.docx]

**Search strategies for Phase 1 and 2 in MEDLINE**

Phase 1

Database(s): **Ovid MEDLINE(R) ALL**1946 to November 28, 2023
Search Strategy:

| **#** | **Searches** |
| --- | --- |
| 1 | ("aged person" or elder* or older or senior or "old age" or "old people" or "older person" or geriatric or retire* or "advanced age population").ab,kw,ti. |
| 2 | exp Aged/ |
| 3 | (nutrition* or diet* or food or fruit* or vegetable* or protein or vitamin or supplement* or micronutrient* or macronutrient*).ab,kw,ti. |
| 4 | exp Diet, Healthy/ or exp "Diet, Food, and Nutrition"/ or exp Diet/ |
| 5 | exp Dietary Supplements/ |
| 6 | ("nutritional status" or anaemia or anemia or malnutrition or "nutritional assessment" or malnourished or undernutrition or obesity or overweight or undernutrition or overnutrition).ab,kw,ti. |
| 7 | ("food insecurity" or "food security" or hunger or "food consumption" or "food deprivation" or "food supply").ab,kw,ti. |
| 8 | "systematic review".pt. or "Systematic Reviews as Topic"/ or "Cochrane Database of Systematic Reviews".jn. or (evidence report technology assessment or evidence report technology assessment summary).jn. or (((comprehensive* or integrative or mapping or rapid or realist or scoping or systematic or systematical or systematically or systematicaly or systematicly or umbrella) adj3 (bibliographical or bibliographically or bibliographics or literature or review or reviews)) or (state adj3 art adj1 review) or (research adj2 synthesis) or ((data or information) adj3 synthesis)).ti,ab,kf. or ((data adj2 (extract or extracting or extractings or extraction or extraction)).ti,ab,kf. and ("review".ti. or "review".pt.)) or (((electronic or searched) adj2 database*) and (eligibility or excluded or exclusion or included or inclusion)).ti,ab,kf. or (overview adj4 reviews).ti,ab,kf. or ((review adj3 (rationale or evidence)).ti,ab. and "review".pt.) or (PRISMA or (preferred adj1 reporting)).ab. or (cinahl or (cochrane adj3 (trial or trials)) or embase or medline or psyclit or (psycinfo not (psycinfo adj1 database)) or pubmed or scopus or (sociological adj1 abstracts) or (web adj2 science)).ab. |
| 9 | "narrative review".ab,kw,ti. |
| 10 | "scoping review".ab,kw,ti. |
| 11 | exp Africa, Western/ or exp Africa, Central/ or exp "Africa South of the Sahara"/ or exp Africa/ or exp Africa, Northern/ or exp Africa, Eastern/ or exp Africa, Southern/ |
| 12 | 1 or 2 |
| 13 | 3 or 4 or 5 or 6 or 7 |
| 14 | 8 or 9 or 10 |
| 15 | (Algeria or Angola or Benin or Botswana or "Burkina Faso" or Burundi or Cameroon or "Cape Verde" or "Central African Republic" or Chad or Comoros or Congo or Djibouti or Egypt or "Equatorial Guinea" or Eritrea or Ethiopia or Gabon or Gambia or Ghana or Guinea or "Guinea Bissau" or "Ivory Coast" or "Cote D'Ivoire" or Kenya or Lesotho or Liberia or Libya or Madagascar or Malawi or Mali or Mauritania or Mauritius or Morocco or Mozambique or Namibia or Niger or Nigeria or Reunion or Rwanda or "Sao Tome" or Senegal or Seychelles or "Sierra Leone" or Somalia or "South Africa" or Sudan or Eswatini or Swaziland or Tanzania or Togo or Tunisia or Uganda or "Western Sahara" or Zaire or Zambia or Zimbabwe).ab,kw,ti. |
| 16 | 11 or 15 |
| 17 | 12 and 13 and 14 and 16 |

Phase 2

Database(s): **Ovid MEDLINE(R) ALL**1946 to February 05, 2024
Search Strategy:

| **#** | **Searches** |
| --- | --- |
| 1 | (nutrition* or diet* or food or fruit* or vegetable* or protein or vitamin or supplement* or micronutrient* or macronutrient*).ab,kw,ti. |
| 2 | exp Diet, Healthy/ or exp "Diet, Food, and Nutrition"/ or exp Diet/ |
| 3 | exp Dietary Supplements/ |
| 4 | ("nutritional status" or anaemia or anemia or malnutrition or "nutritional assessment" or malnourished or undernutrition or obesity or overweight or undernutrition or overnutrition).ab,kw,ti. |
| 5 | ("food insecurity" or "food security" or hunger or "food consumption" or "food deprivation" or "food supply").ab,kw,ti. |
| 6 | (promotion or prevention or intervention or program or programme or plan or "health promotion" or campaign* or "health programme*" or "health program*" or "health prevention" or "health education" or "health literacy" or education or "health communication" or "health advocacy" or "community advocacy" or "social campaign*" or "health coaching" or "environmental change strateg*" or "health* environment" or "community mobili#ation" or "behavior modification" or "behaviour modification" or screen* or "primary prevention" or "health screen*" or "social network" or "health change*" or "legislation" or "regulation" or "cash transfer*" or "non-contributory pension*" or "enterprise grant*" or welfare or "financial support" or "social security").ab,kw,ti. |
| 7 | controlled clinical trial.pt. |
| 8 | randomized controlled trial.pt. |
| 9 | clinical trials as topic/ |
| 10 | (randomi#ed or randomi#ation or randomi#ing).ti,ab,kf. |
| 11 | (RCT or "at random" or (random* adj3 (administ* or allocat* or assign* or class* or cluster or crossover or cross-over or control* or determine* or divide* or division or distribut* or expose* or fashion or number* or place* or pragmatic or quasi or recruit* or split or subsitut* or treat*))).ti,ab,kf. |
| 12 | placebo.ab,ti,kf. |
| 13 | trial.ti. |
| 14 | (control* adj3 group*).ab. |
| 15 | (control* and (trial or study or group*) and (waitlist* or wait* list* or ((treatment or care) adj2 usual))).ti,ab,kf. |
| 16 | ((single or double or triple or treble) adj2 (blind* or mask* or dummy)).ti,ab,kf. |
| 17 | double-blind method/ or random allocation/ or single-blind method/ |
| 18 | 1 or 2 or 3 or 4 or 5 |
| 19 | 7 or 8 or 9 or 10 or 11 or 12 or 13 or 14 or 15 or 16 or 17 |
| 20 | exp Africa, Western/ or exp Africa, Northern/ or exp South Africa/ or exp Africa, Eastern/ or africa.mp. or exp "Africa South of the Sahara"/ or exp Africa, Central/ or exp Africa/ or exp Africa, Southern/ |
| 21 | (Algeria or Angola or Benin or Botswana or "Burkina Faso" or Burundi or Cameroon or "Cape Verde" or "Central African Republic" or Chad or Comoros or Congo or Djibouti or Egypt or "Equatorial Guinea" or Eritrea or Ethiopia or Gabon or Gambia or Ghana or Guinea or "Guinea Bissau" or "Ivory Coast" or "Cote D'Ivoire" or Kenya or Lesotho or Liberia or Libya or Madagascar or Malawi or Mali or Mauritania or Mauritius or Morocco or Mozambique or Namibia or Niger or Nigeria or Reunion or Rwanda or "Sao Tome" or Senegal or Seychelles or "Sierra Leone" or Somalia or "South Africa" or Sudan or Eswatini or Swaziland or Tanzania or Togo or Tunisia or Uganda or "Western Sahara" or Zaire or Zambia or Zimbabwe).ab,kw,ti. |
| 22 | exp Aged/ |
| 23 | ("aged person" or elder* or older or senior or "old age" or "old people" or "older person" or geriatric or retire* or "advanced age population").ab,kw,ti. |
| 24 | 20 or 21 |
| 25 | 22 or 23 |
| 26 | 6 or 19 |
| 27 | 18 and 24 and 25 and 26 |
| 28 | (nutrition* or diet* or food or fruit* or vegetable* or protein or vitamin or supplement* or micronutrient* or macronutrient*).ab,kw,ti. |
| 29 | exp Diet, Healthy/ or exp "Diet, Food, and Nutrition"/ or exp Diet/ |
| 30 | exp Dietary Supplements/ |
| 31 | ("nutritional status" or anaemia or anemia or malnutrition or "nutritional assessment" or malnourished or undernutrition or obesity or overweight or undernutrition or overnutrition).ab,kw,ti. |
| 32 | ("food insecurity" or "food security" or hunger or "food consumption" or "food deprivation" or "food supply").ab,kw,ti. |
| 33 | (promotion or prevention or intervention or program or programme or plan or "health promotion" or campaign* or "health programme*" or "health program*" or "health prevention" or "health education" or "health literacy" or education or "health communication" or "health advocacy" or "community advocacy" or "social campaign*" or "health coaching" or "environmental change strateg*" or "health* environment" or "community mobili#ation" or "behavior modification" or "behaviour modification" or screen* or "primary prevention" or "health screen*" or "social network" or "health change*" or "legislation" or "regulation" or "cash transfer*" or "non-contributory pension*" or "enterprise grant*" or welfare or "financial support" or "social security").ab,kw,ti. |
| 34 | controlled clinical trial.pt. |
| 35 | randomized controlled trial.pt. |
| 36 | clinical trials as topic/ |
| 37 | (randomi#ed or randomi#ation or randomi#ing).ti,ab,kf. |
| 38 | (RCT or "at random" or (random* adj3 (administ* or allocat* or assign* or class* or cluster or crossover or cross-over or control* or determine* or divide* or division or distribut* or expose* or fashion or number* or place* or pragmatic or quasi or recruit* or split or subsitut* or treat*))).ti,ab,kf. |
| 39 | placebo.ab,ti,kf. |
| 40 | trial.ti. |
| 41 | (control* adj3 group*).ab. |
| 42 | (control* and (trial or study or group*) and (waitlist* or wait* list* or ((treatment or care) adj2 usual))).ti,ab,kf. |
| 43 | ((single or double or triple or treble) adj2 (blind* or mask* or dummy)).ti,ab,kf. |
| 44 | double-blind method/ or random allocation/ or single-blind method/ |
| 45 | 28 or 29 or 30 or 31 or 32 |
| 46 | 34 or 35 or 36 or 37 or 38 or 39 or 40 or 41 or 42 or 43 or 44 |
| 47 | exp Africa, Western/ or exp Africa, Northern/ or exp South Africa/ or exp Africa, Eastern/ or africa.mp. or exp "Africa South of the Sahara"/ or exp Africa, Central/ or exp Africa/ or exp Africa, Southern/ |
| 48 | (Algeria or Angola or Benin or Botswana or "Burkina Faso" or Burundi or Cameroon or "Cape Verde" or "Central African Republic" or Chad or Comoros or Congo or Djibouti or Egypt or "Equatorial Guinea" or Eritrea or Ethiopia or Gabon or Gambia or Ghana or Guinea or "Guinea Bissau" or "Ivory Coast" or "Cote D'Ivoire" or Kenya or Lesotho or Liberia or Libya or Madagascar or Malawi or Mali or Mauritania or Mauritius or Morocco or Mozambique or Namibia or Niger or Nigeria or Reunion or Rwanda or "Sao Tome" or Senegal or Seychelles or "Sierra Leone" or Somalia or "South Africa" or Sudan or Eswatini or Swaziland or Tanzania or Togo or Tunisia or Uganda or "Western Sahara" or Zaire or Zambia or Zimbabwe).ab,kw,ti. |
| 49 | exp Aged/ |
| 50 | ("aged person" or elder* or older or senior or "old age" or "old people" or "older person" or geriatric or retire* or "advanced age population").ab,kw,ti. |
| 51 | 47 or 48 |
| 52 | 49 or 50 |
| 53 | 33 or 46 |
| 54 | 45 and 51 and 52 and 53 |

**Supplementary Table 1: Citations of reviews reporting on factors associated with nutrition outcomes and dietary practices**

| Theme | Associated factor | Evidence sources |
| --- | --- | --- |
| Malnutrition | Low socio-economic status | [34, 44] |
|  | Rural living | [44] |
|  | Appetite and digestion problems | [44] |
|  | Poor mental health | [34] |
|  | Food choices | [32, 44] |
| Food insecurity | Disability | [39] |
|  | Low socioeconomic status | [39] |
|  | Poor mental health | [39] |
|  | Living alone | [39] |
|  | Rural living | [39, 43] |
|  | Larger household size | [39, 43] |
|  | No pension or grant | [39] |
| Food choices | Appetite | [43] |
|  | Knowledge | [45] |
|  | Routine | [45] |
|  | Taste | [45] |
|  | Digestion problems | [43] |
|  | Company during mealtimes | [45] |
|  | Media | [45] |
|  | Culture | [45] |
|  | Food availability | [45] |
| Recommended dietary intake* | Urban living | [43] |
|  | Rural living | [43] |
|  | High socioeconomic status | [43, 46] |
| Obesity | Urban living | [43, 44] |
|  | Being female | [43, 44] |
|  | High socioeconomic status | [44] |
| *Two reviews reported recommended fruit and vegetable intake while one review reported zinc intake | | |
